# Differential Transcriptome Profile of Peripheral White Cells to Identify Biomarkers Involved in Oxaliplatin Induced Neuropathy

**DOI:** 10.3390/jpm4020282

**Published:** 2014-06-05

**Authors:** Manuel Morales, Julio Ávila, Rebeca González-Fernández, Laia Boronat, María Luisa Soriano, Pablo Martín-Vasallo

**Affiliations:** 1 Service of Oncology, Hospital Universitario Nuestra Señora de Candelaria, Santa Cruz de Tenerife, 38010 Tenerife, Spain; E-Mails: mmoraleg@ull.es (M.M.); laiamed@hotmail.com (L.B.); mariamsori@hotmail.com (M.L.S.); 2 Service of Medical Oncology, Hospiten Rambla, Hospiten Hospitals Group, Santa Cruz de Tenerife, 38001 Tenerife, Spain; 3 Developmental Biology Laboratory, Department of Biochemistry and Molecular Biology, University of La Laguna, Av. Astrofísico Sánchez s/n, 38206 La Laguna, Spain; E-Mails: javila@ull.es (J.Á.); refernan@ull.es (R.G.-F.)

**Keywords:** transcriptome profile, chemotherapy, oxaliplatin, neuropathy, FOLFOX, CAPOX, colon-adenocarcinoma

## Abstract

Anticancer chemotherapy (CT) produces non-desirable effects on normal healthy cells and tissues. Oxaliplatin is widely used in the treatment of colorectal cancer and responsible for the development of sensory neuropathy in varying degrees, from complete tolerance to chronic neuropathic symptoms. We studied the differential gene expression of peripheral leukocytes in patients receiving oxaliplatin-based chemotherapy to find genes and pathways involved in oxaliplatin-induced peripheral neuropathy. Circulating white cells were obtained prior and after three cycles of FOLFOX or CAPOX chemotherapy from two groups of patients: with or without neuropathy. RNA was purified, and transcriptomes were analyzed. Differential transcriptomics revealed a total of 502 genes, which were significantly up- or down-regulated as a result of chemotherapy treatment. Nine of those genes were expressed in only one of two situations: *CSHL1*, *GH1*, *KCMF1*, *IL36G* and *EFCAB8* turned off after CT, and *CSRP2*, *IQGAP1*, *GNRH2*, *SMIM1* and *C5orf17* turned on after CT. These genes are likely to be associated with the onset of oxaliplatin-induced peripheral neuropathy. The quantification of their expression in peripheral white cells may help to predict non-desirable side effects and, consequently, allow a better, more personalized chemotherapy.

## 1. Introduction

Oxaliplatin (OX), a part of FOLFOX chemotherapy (FOL—Folinic acid, leucovorin, F—Fluorouracil, 5-FU) or CAPOX (CA—capecitabine, OX—oxaliplatin) chemotherapy is a widely used anticancer chemotherapy (CT)-agent for colorectal cancer. Oxaliplatin is responsible for unpredictable peripheral neural toxicity (polyneuropathy) [1]. Wide differences in susceptibility among patients have been reported ranging from complete tolerance to two years of neuropathic symptoms [2,3]. Neurological symptoms should be taken into account to adapt oxaliplatin dosing in order to prevent neuropathy. However, often, reduction of oxaliplatin dose is applied late and, consequently, not only is neuropathy not avoided but the efficacy of oxaliplatin is also diminished [4,5,6]. FOLFOX or CAPOX-CT varies the expression of some genes, which ultimately drives the cell to one of two opposite fates: survival, mitosis, and heterogeneity or apoptosis. Along with these effects on cancer cells, some of these variations could be causative of CT unwanted effects.

There is a need for non-invasive, sensitive and specific biomarkers, which will allow patients at risk for oxaliplatin-induced neuropathy to prevent the occurrence of long term toxicity or permanent damage.

In the current study, we performed a genome-wide association analysis in a cohort of patients with colon cancer who received oxaliplatin-based combination chemotherapy to try to identify genes associated with severe oxaliplatin-induced chronic peripheral neuropathy (OXPN). In addition, this approach can be used to gain information about genes and specific signal transduction pathways involved in peripheral neural toxicity induced by oxaliplatin.

Furthermore, the knowledge of selected markers may provide additional information about the differences between patients more or less likely to develop neuropathy. Subsequently, this information can be taken into account for alternative colorectal cancer treatment protocols.

## 2. Results

### 2.1. Patients and Clinical Evaluation of Neurotoxicity

Differential transcriptomics results were obtained from RNA from six selected patients from a total pool of 27. None of those six presented any kind of neuropathy previous to FOLFOX/CAPOX-CT. Table 1 shows the relevant toxicities of the patients.

**Table 1:** Patients selected for this study and their grade of toxicities.

Patients	Anemia	Thrombopenia	Neutropenia	Neurotoxicity	Mucositis
**ASC**	Grade I	No	No	Grade III	No
**LGM**	Grade II	No	No	Grade II	No
**SDB**	No	Grade II	No	Grade III	No
**ICHB**	Grade II	Grade I	No	Grade I	Grade I
**ASA**	No	Grade I	No	Grade I	No
**AGS**	No	Grade I	No	Grade I	No

Peripheral neuropathy was evaluated and graded by oncologists on Day 1 of each CT cycle according to the National Cancer Institute Common Terminology Criteria for Adverse Events (NCI-CTCAE), version 2.0 from January 2004 to August 2006, and version 3.0 from August 2006 onwards.

The oxaliplatin dose was reduced from 130 to 100 mg/m^2^ and from 85 to 75 mg/m^2^ for patients treated with CAPOX and FOLFOX regimens, respectively, in cases of prolonged (≥7 but <14 days) Grade 2 or temporary (<7 days) Grade 3 neuropathy.

### 2.2. Analysis of Transcriptomics

Differential transcriptomics showed a series of genes expressed at variable numbers of mRNA copies ranging from a 18.78- to −53.34-fold change post- *versus* pre-CT treatment. Comparison of both series produced a list of 502 genes shown as supplementary data along with statistical significance based on the amount of mRNA copies specific for each gene pre- and post CT. A total of 363 genes was expressed, at least twice as much as in the other series.

**Figure 1:**
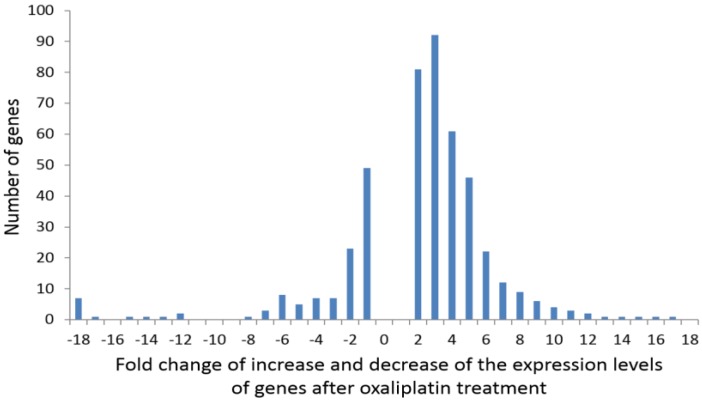
Distribution of frequencies of the genes whose expression showed a given-fold increase or decrease after oxaliplatin treatment.

Figure 1 shows the distribution of frequencies of the genes whose expression changed by a given-fold increase or decrease after oxaliplatin treatment. Among others, the list includes those genes coding for metabolic proteins (mitochondrial and cytosolic), cellular response to organic substance (such as *PPARG*, *FGF2*, *EREG*, *MIF*, *SERPINE1*, *BMPR1B*, *LMNA*), cellular component movement (such as *HES1*, *KIF2C*, *NRP1*, *ITGA11*, *FSCN1*, *PODXL*, *SLC7A5*, *EGFR*, *CDH2*), response to drugs (such as *SLC22A1*, *PPARG*, *SOD1-2*, *SLC1A3*, *ALDH3A1*, *ATP1A3*, *PTGS2*) and cell morphogenesis involved in differentiation (such as *HES1*, *DRAXIN*, *NRP1*, *SLC1A3*, *WNT5A-7B*, *COL4A1-3A1-4A2*, *TGFB1I1*, *BMP4*). 

### 2.3. Characteristics of Genes with “On-Off” Differential Expression

“On-off” differential expression genes were those expressed at a certain level prior to CT with no expression at all after three cycles or, the other way around, those genes showing no expression before patients underwent CT that reached a certain expression level after CT.

mRNA copies of *CSHL1*, *GH1*, *KCMF1*, *IL36G*, and *EFCAB8* genes were found in white cells previous to CT, however, after three FOLFOX/CAPOX cycles, no message for these genes was detected. On the other hand, *CSRP2*, *IQGAP1*, *GNRH2*, *C5orf17* and *SMIM1* genes did not show any expression in white cells pre-chemotherapy, but after CT, mRNA specific to them was found. Table 2 shows quantitative characteristics of these facts.

**Table 2:** Genes whose expression varied “on-off” after oxaliplatin chemotherapy. Relative expression levels, PRE, stands for expression level pre-chemotherapy and POST for post-chemotherapy. LMNA and DLEU7 are genes with polymorphisms sensitive to oxaliplatin that overlap our study.

Gene	Genbank Acc. Number	PRE	POST
Gonadotropin-releasing hormone 2 (*GNRH2*)	NM_001501.1	0	5.8
Cysteine and glycine-rich protein 2 (*CSRP2*)	NM_001321.1	0	7.8
C5orf17 chromosome 5 open reading frame 17 (*C5orf17*)	NC_000005.9	0	8.8
IQ motif containing GTPase activating protein 1 (*IQGAP1*)	NM_003870.3	0	229.5
EF-hand calcium binding domain 8 (*EFCAB8*)	XM_006723897.1	4.1	0
Chorionic somatomammotropin hormone-like 1 (*CSHL1*)- Growth hormone 1 (*GH1*)	NM_001318.2- NM_000515.3	7.2	0
Small integral membrane protein 1 (*SMIM1*)	NM_001163724.2	8.2	0
Potassium channel modulatory factor 1 (*KCMF1*)	NM_020122.4	12.3	0
Interleukin 36, gamma (*IL36G*)	NM_001278568.1	13.4	0
Lamin A/C (*LMNA*)	NM_001257374.2	325	1075.5
Deleted in lymphocytic leukemia 7 (*DLEU7*)	NM_198989.2	25.7	3.9

### 2.4. Interactions among Proteins Coded by Differentially Expressed Genes

Based on these experimental data, we have elaborated the most significant network groups of differentially regulated genes associated with OXPN. These groups were generated on the basis of the protein-protein interaction study using the databases and programs available [7,8,9].

## 3. Discussion

Cytotoxic CT affects the DNA of both tumor and normal cells. This effect is responsible for the therapeutic effect of CT, but also for its toxicity. CT-induced toxicity can be reversible, (alopecia, hematologic or digestive toxicity) or chronic. One of the long lasting side effects of CT is peripheral neuropathy, which affects 30% of patients treated with chemotherapy based on platinum compounds and paclitaxel. Neuropathy is a dose-limiting side effect of chemotherapy that causes worsening of the quality of life and leads to 20% of patients having to stop treatment. In many patients, despite treatment cessation, peripheral neuropathy worsens [10].

Another important consequence of the effect of CT on DNA is the development of secondary tumors or meylodysplastic syndromes [11].

The aim of our study is to observe the effects of CT on the gene expression patterns in leukocytes and to find out whether specific alterations of gene expression are related with the development of neuropathy. Bone marrow is one of the organs most affected by CT. The effects of CT on the DNA of bone marrow stem cells, with the consequent gene expression patterns, can be detected in circulating leukocytes. Alterations in gene expression observed in leukocytes could reflect the effects of CT on other target cells. Pharmacogenomics uses genome-wide studies to establish loci for drug responses and toxicity. Genome-wide polymorphism studies describe variation between individuals and associate these with response and toxicity [12,13,14,15]. The detection of different single nucleotide polymorphisms (SNPs) in enzymes involved in the metabolism, absorption, distribution, and excretion of certain drugs can be used to identify patients with a higher probability of developing certain toxicities, including neurologic toxicity [16].

Our present approach tries to detect drug-related alterations in gene expression that can be associated with the development of CT-induced neuropathy. In contrast to the SNPs, which are genetically determined, the continued exposure of cells to genotoxic drugs can modify the gene expression affecting the cell’s physiology. Some of these gene expression patterns can be adaptive mechanisms of the cells to the cytotoxic agent while others can be induced by the toxic effect of the drugs on nuclear or mitochondrial DNA. Another objective of our research is to determine if these alterations in gene expression are transitory or permanent. This is an issue of further research. The transitory alterations could reflect adaptive changes to the drugs, whereas the permanent changes could reflect damage to the genetic material that could help explain some long lasting effects or consequences of chemotherapy such as asthenia, anemia, neuropathy, myelodisplastic syndromes and second neoplasias. In a further step, we started another study to determine whether these alterations can be detected in tumors after the administration of neoadjuvant chemotherapy and whether these alterations have any prognostic implications.

### Some Characteristics and Comments on Genes with “On-Off” Variations in this Study

***CSHL1*, *GH1*** (chorionic somatomammotropin hormone-like 1, growth hormone 1). This gene is one of the four members forming the somatotropin/prolactin family coding for protein hormones, which play an important role in growth control [NCBI, HGNC]. An association exists between GH expression with lymph node metastasis, tumor stage, and the proliferative index in mammary carcinomas and with ovarian metastases in endometrial carcinomas [17].

***KCMF1*** (potassium channel modulatory factor 1, also known as FIGC (bFGF-induced in gastric cancer)) is a 381 amino-acid protein-coding gene expressed at different levels in virtually all tissues [18]. The designation “potassium channel modulatory factor” is somewhat misleading. To the best of our knowledge, we are not aware of any functional or structural evidence that links KCMF1 to potassium channel signaling. *KCMF1* protein encodes two N-terminal zinc-finger domains (ZZ-type), a coiled-coil domain and a C-terminal proline-rich region. In addition, a putative nuclear import signal and two leucine-rich nuclear export signals have been identified on the basis of sequence analysis [19]. *KCMF1* has been reported to be down-regulated in Ewing’s sarcoma cell lines after the over expression of CD99 and up-regulated in gastric cancer (*KCMF1* as a CD99-regulated putative metastasis suppressor gene) [20].

However, the temporal and spatial expression of *KCMF1* preneoplastic lesions of pancreatic cancer in human and murine carcinoma argues against a tumor-suppressive function of KCMF1 *in vivo* [21].

Beilke *et al*. [19] showed that the nuclear zinc finger protein *KCMF1* was over-expressed in epithelial cancers, and especially in human and mouse pancreatic cancer. *KCMF1* enhanced proliferation, migration and invasion. The down-regulation of *KCMF1 in vivo* reduced preneoplastic changes in the transforming growth factor-α transgenic pancreatic cancer model.

***EFCAB8*** (EF-Hand Calcium Binding Domain 8). The only information available about this domain is in relation to its annotation in the human genome project and gene association with inattention and hyperactivity-impulsivity and kidney disease in type 1 diabetes [22,23,24].

***Q9NZH8*** (*IL36G_HUMAN*) or Interleukin-36 gamma is an IL-1 family member that signal through the IL-1 receptor family members [25]. It is highly expressed in tissues containing epithelial cells: skin, lung, stomach and esophagus. IL-36γ has the capability to establish an inflammatory gene expression profile in human primary keratinocytes [26]. Il36γ functions as an agonist of NF-kappa B activation through the orphan IL-1-receptor-related protein 2/IL1RL2 [27] is a part of a signaling system analogous to that present in epithelial barriers and takes part in the local inflammatory response.

***CSRP2***, also known as *CRP2*, codes for cysteine and glycine-rich protein 2, a member of the *CSRP* family of genes, encoding a group of LIM domain proteins. *CRP2* contains two copies of the cysteine-rich amino acid sequence motif (LIM) with putative zinc-binding activity (NCBI). *CRP2* is proposed to function as a molecular adapter involved in regulating ordered cell growth, development, and cellular differentiation in the liver [28] and shows an increased expression during dedifferentiation of hepatocellular carcinoma [29].

***IQGAP1***, or p195, IQ motif containing the GTPase activating protein 1, “IQ” refers to the first two amino acids of the motif, isoleucine and glutamine. *IQGAP1*, ubiquitously expressed, has been related with cellular complex processes such as transcription, cell adhesion, organization of the actin cytoskeleton, and regulation of the cell cycle, thereby serving as a scaffold to integrate signaling pathways [30,31]. It is interesting to point out the fact that expression of the protein is up-regulated by gene amplification in several cell lines and its over-expression and distinct membrane localization is also observed in a range of tumors, moreover, about 10% of genes that show increased expression in metastatic cells are *IQGAP1*-binding partners [32,33]. Because *IQGAP1* plays a significant role in the propagation of the MAPK signaling pathway through the RAS-RAF-MEK-ERK signaling kinase pathway involved in tumor cell proliferation and survival, *IQGAP1* has been the focus of intense cancer drug development efforts [34,35,36].

***SMIM1***. Small Integral Membrane Protein 1 is responsible for the Vel blood group system. SMIM1 is expressed at high levels in bone marrow and erythroleukemia cell lines and at lower levels in non-hematopoietic tissues. The Vel antigen is present on red blood cells (RBCs) of all humans except for rare Vel-negative individuals who can form antibodies to Vel in response to transfusion or pregnancy. These antibodies may cause severe hemolytic reactions in blood recipients [37,38,39].

***C5orf17***.The so far only reference to this gene refers to one of the sixty-one interacting cell proteins detected to interact with the latency-associated nuclear antigen (LANA) of the Kaposi’s sarcoma-associated herpesvirus [40].

When the differentially expressed genes found in this study where compared with those previously described in the literature as polymorphisms of genetic predictors for severe oxaliplatin-induced peripheral neuropathy (around fifty genes previously reported), only *DLEU7* (deleted in lymphocytic leukemia 7) and *Lamin A/C* (which encodes a nuclear envelope protein) were found to significantly vary their expression levels after CT [12,15]. These genes are also included in Table 2. A genome-wide association analysis of patients with colon cancer who received oxaliplatin-based chemotherapy associated a single nucleotide polymorphism (C/G) of DLEU7 with a higher possibility of suffering from polyneuropathy [15]. The mutation (c.892C>T–p.R298C) in the gene coding for Lamin A/C leads to the motor and sensory neuropathy Charcot–Marie–Tooth Type 2 [12]. Further studies in both genes need to be performed in white cells and nerve samples in order to establish the association of oxaliplatin-gene-neuropathy.

The role of the previously-described genes in peripheral neuropathy are not obvious. Referred genes could either be responsible for the pathogenesis of neuropathy following the “stimulus” embodied by oxaliplatin or could be up- or down-regulated as an adaptive process.

**Figure 2:**
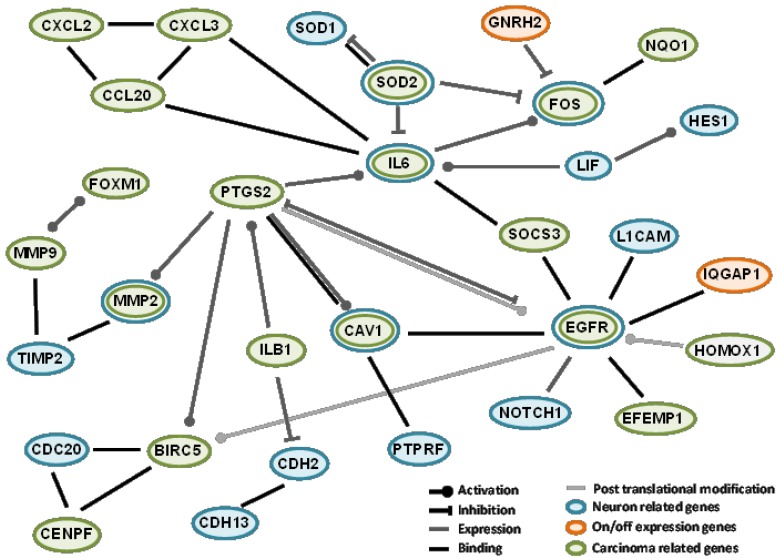
Protein interaction and association network among some of the genes identified in the differential transcriptomics. Orange ovals represent genes with “on-off” differential expression, green oval genes relate with breast neoplasms and carcinoma hepatocellular and blue oval genes relate to neurogenesis, neuron differentiation, and neuron development.

As a first approach, to better understand the involvement of differentially expressed genes in neuropathy, protein interactions among “on-off” differentially expressed genes and their association with several networks were analyzed. In order to generate an OXPN-network, from all the networks analyzed, we selected those involving genes associated with neurogenesis, neuron differentiation, and neuron development and, related to cancer, those associated with breast neoplasms and hepatocellular carcinoma (Figure 2). We selected these networks because they were the more closely related to our field of study, given the (fact of a) lack of networks for any peripheral neuropathy or colon carcinoma among those suggested by the transcriptome presented in our work. We report interactions only for IQGAP1 and GNRH2 because no interactions between the other found genes and cancer or neuropathy are available through the databases mentioned in the Experimental Section of study.

To generate the OXPN-network, we took into account several facts, for instance, that IQGAP1 binds to EGFR and GNRH2 inhibits *fos* gene expression [41,42]. EGFR and fos are involved in biological processes including neurogenesis, neuron differentiation, and neuron development, and also breast neoplasm and hepatocellular carcinoma. EGFR expression increases after CT, while *fos* gene expression is diminished. “fos” is part of the activator protein-1 (AP-1) transcription factor implicated in the regulation of cell proliferation, differentiation, and transformation [43]. In the brain, AP-1 transient activation is related to neuronal plasticity [44,45]. IQGAP1 has been proposed as a modulator of EGFR receptor activation [41]. Activated EGFR recruits downstream signaling molecules, leading to the activation of different pathways important in tumor growth, progression, and survival [46,47]. Moreover, EGFR nuclear accumulation has been linked to malignant phenotypes such as therapeutic resistance [48]. EGFR also plays an important role in nervous system development affecting neural cell survival, proliferation, and differentiation [49,50,51]. In the network we show that EGFR also associates with other proteins implicated in this process such us L1CAM, a cell adhesion molecule involved in neuron-neuron adhesion and neurite fasciculation [52,53], BIRC5, a member of the inhibitor of apoptosis (IAP) gene family that prevents apoptosis [54,55], and EFEM1, an extracellular matrix glycoprotein with a possible role in tumor aggressiveness [56,57,58].

Complete transcriptomics is a powerful tool to get a huge amount of data regarding the commitment of gene expression at a given circumstance of the cell. A big selection has been made in steps reported in this study. A total of 502 genes of the whole human genome has been pointed out, and some of these selected genes are most probably associated with the clinical outcome of OXPN. The quantification of their expression in peripheral white cells may help to predict non-desirable side effects and, consequently, set a better personalized CT. Because the white cell is not the real target in neuropathy, we have to be cautious with the interpretation of our results and try to find common reporters in neural cells and white cells in order to get information about the neuropathy process with the least invasive procedure. Current studies in our laboratory confirm patient by patient at the mRNA (qRT-PCR) and protein (immunohistochemistry) levels that some the genes reported here are expressed in white cells. However, the selection of patients and the gap between treatments makes this study proceed slower than desired. Furthermore, studies of expression of these genes at the protein level in tumor samples, in the pre- and post-CTsituations, will help to study the effects of CT on gene expression in the tumor cells and, consequently, could help to better understand the mechanisms of cytotoxicity, drug resistance, and tumor progression.

## 4. Experimental

### 4.1. Patients

This study was approved by the Ethics Committee of the Universidad de La Laguna and Ethical Committee of the Hospital Universitario Nuestra Señora de Candelaria. All the patients signed informed-consent documents before entering the project. All the study subjects were of whites skin color and of European origin, recruited from the reference medical area of the hospital. All patients were treated with FOLFOX (oxaliplatin 100 mg/m^2^ iv over 2 h, day 1; leucovorin calcium 400 mg/m^2^ iv over 2 h, day 1; followed by 5-fluorouracil 400 mg/m^2^ iv bolus and followed by 5-fluorouracil 2400 mg/m^2^ iv over 46 h; every 14 days) or CAPOX (oxaliplatin 130 mg/m^2^ iv over 2 h, day 1 and capecitabine 1000 mg/m^2^ twice daily from evening d1 to morning day 15; every 21 days)—CT, and no patient had received any previous CT or radiation therapy.

### 4.2. Leukocytes Isolation

Blood samples were withdrawn immediately before patients underwent the first FOLFOX dose and after three cycles (at the time the fourth cycle was ready to be administrated). A volume of PBS 1× (NaH_2_PO_4_ 1.9 mM, Na_2_HPO_4_ 8.1 mM, NaCl 154 mM) was added to an identical volume of freshly collected blood and mixed gently (usually 5 mL each). The mix was placed over 5 mL Ficoll-Hypaque cushion (d = 1.077 g/mL) (Sigma, St Louis, MO, USA) and centrifuged for 30 min, at 800 *g*. The intermediate layer formed by mononuclear white cells was gently aspirated and washed three times in three volumes of HBSS (KCl 5.4 mM, Na_2_HPO_4_ 0.3 mM, KH_2_PO_4_ 0.4 mM, NaHCO_3_ 4.2 mM, CaCl_2_ 1.3 mM, MgCl_2_ 0.5 mM, MgSO_4_ 0.6 mM, NaCl 137 mM, D-glucose 5.6 mM, phenol red 0.02%), centrifuged at 300 *g* for 10 min. The pellet-containing cells were resuspended in one ml of PBS. Trypan blue test and cell counting in Neubauer chamber was performed afterwards. The amount of collected cells ranged from 3 to 4 × 10^6^ cells.

### 4.3. White Cell mRNA Extraction

Collected white cells from 5 mL blood were spun at 4000 *g* for 5 min, mRNA from the cellular pellet was obtained with the “Total Aurum RNA extraction kit” (Bio-Rad Laboratories, Hercules, CA, USA), following the manufacturer’s instructions. The mRNA obtained was diluted in a final volume of 50 µL.

### 4.4. RNA-Seq

PolyA-RNA was isolated from 10 micrograms of total RNA using the MicroPoly(A) Purist kit™ (Ambion, Austin, TX, USA). Total PolyA-RNA samples were used to generate whole transcriptome libraries for sequencing on the SOLiD 5500XL platform, following the manufacturer’s recommendation (Life Technologies, Foster City, CA, USA). No RNA-spike in controls was used. Amplified cDNA quality was analyzed by the Bioanalyzer 2100 DNA 1000 kit (Agilent Technologies, Madrid, Spain) and quantified using the Qubit 2.0 Fluorometer (Invitrogen, Carlsbad, CA, USA). The whole transcriptome libraries were used for making SOLiD templated beads following the SOLiD Templated Bead Preparation guide. Bead quality was estimated based on WFA (workflow analysis) parameters. The samples were sequenced using the 50625 paired-end protocol, generating 75 nt + 35 nt (Paired-End) + 5 nt (Barcode) sequences. Quality data were measured using software SETS parameters (SOLiD Experimental Tracking System).

### 4.5. Computational Analysis of RNA-Seq Data

The initial whole transcriptome paired-end reads obtained from sequencing were mapped against the latest version of the human genome (version GRchr37/hg19) using the RNA Transcript Mapping with New Suffix Array Algorithm [59]. It was using the standard Bioscope software parameters of version 1.3, in paired ends and for the whole transcriptome analysis. For both reads, forwards and reverse, the seed was the first 25 nucleotides with a maximum of two mismatches allowed. The aligned records were reported in BAM/SAM format [60]. Bad quality reads (Phred score < 10) were eliminated using Picard Tools software, version 1.83 [61]. 

Subsequently, isoform and gene prediction, and differential expression were estimated using the cufflinks method version 2.02 [62]. This method relies on different normalized processes based on the depth of the global samples, CG composition, and length of genes. In the differential expression process, this method relies on a Poisson model to estimate the variance of the RNA-seq data for differential expressions. Finally, genes and isoforms were selected with a p-value adjusted by FDR of less than 0.05 and change threshold of ±2-fold.

### 4.6. Protein-Protein Interactions and Association Study

The predicted protein-protein association and protein-protein interactions study was performed using the database and programs available as STRING [10], IntAct [11] and ConsensusPathDB [12]. Based on the found results, authors elaborated the correspondent figure shown in the Discussion section.

## 5. Conclusions

Quantitation of the expression in peripheral white cells of genes reported in this study is most probably associated to the clinical outcome of oxaliplatin induced peripheral neuropathy. Quantitation of some of these genes may help to predict OXPN and, consequently, set a CT protocol equally efficient but avoiding the peripheral neuropathy side effect.

## Supplementary Files

Supplementary File 1 Supplementary Materials (PDF, 858 KB)
