# Supplementary material for: Differential Transcriptome Profile of Peripheral White Cells to Identify Biomarkers Involved in Oxaliplatin Induced Neuropathy
Source: J Pers Med. 2014 Jun 5;4(2):282–96. doi: 10.3390/jpm4020282 (PMC4263976; doi:10.3390/jpm4020282)
Supplement: Supplementary File 1 — Supplementary Materials (PDF, 858 KB) [file jpm-04-00282-s001.pdf]

Supplementary Material for

Differential Transcriptome Profile of Peripheral White Cells to Identify Biomarkers Involved in Oxaliplatin Induced Neuropathy

Gene	Fold Change	*p*_value	Description
**Genes whose expression increased after three cycles of oxaliplatin therapy (in alphabetical order)**			
A4GALT	5.8	0.017	alpha 1,4-galactosyltransferase [Source:HGNC Symbol;Acc:18149]
AGRN	2.8	0.001	agrin [Source:HGNC Symbol;Acc:329]
AIF1	3.4	0.000	allograft inflammatory factor 1 [Source:HGNC Symbol;Acc:352]
AKAP12	4.5	0.007	A kinase (PRKA) anchor protein 12 [Source:HGNC Symbol;Acc:370]
AKR1C2	8.0	0.006	aldo-keto reductase family 1, member C2 [Source:HGNC Symbol;Acc:385]
ALDH3A1	4.3	0.042	aldehyde dehydrogenase 3 family, member A1 [Source:HGNC Symbol;Acc:405]
AMOTL2	3.5	0.047	angiomotin like 2 [Source:HGNC Symbol;Acc:17812]
ANTXR1	4.5	0.006	anthrax toxin receptor 1 [Source:HGNC Symbol;Acc:21014]
ANXA1	1.7	0.031	annexin A1 [Source:HGNC Symbol;Acc:533]
ANXA2	1.8	0.027	annexin A2 [Source:HGNC Symbol;Acc:537]
AP1M2	3.8	0.026	adaptor-related protein complex 1, mu 2 subunit [Source:HGNC Symbol;Acc:558]
AP2S1	1.7	0.033	adaptor-related protein complex 2, sigma 1 subunit [Source:HGNC Symbol;Acc:565]
ARHGEF17	5.0	0.026	Rho guanine nucleotide exchange factor (GEF) 17 [Source:HGNC Symbol;Acc:21726]
ASGR1	2.9	0.012	asialoglycoprotein receptor 1 [Source:HGNC Symbol;Acc:742]
ASPHD1	7.2	0.028	aspartate beta-hydroxylase domain containing 1 [Source:HGNC Symbol;Acc:27380]
ATP5EP2	5.2	0.003	ATP synthase, H+ transporting, mitochondrial F1 complex, epsilon subunit pseudogene 2 [Source:HGNC Symbol;Acc:34026]
ATP5F1	2.3	0.017	ATP synthase, H+ transporting, mitochondrial Fo complex, subunit B1 [Source:HGNC Symbol;Acc:840]
ATP5I	3.5	0.001	ATP synthase, H+ transporting, mitochondrial Fo complex, subunit E [Source:HGNC Symbol;Acc:846]
ATPIF1	2.0	0.030	ATPase inhibitory factor 1 [Source:HGNC Symbol;Acc:871]

AXL	4.3	0.002	AXL receptor tyrosine kinase [Source:HGNC Symbol;Acc:905]
BASP1	2.1	0.005	brain abundant, membrane attached signal protein 1 [Source:HGNC Symbol;Acc:957]
BCAM	6.6	0.012	basal cell adhesion molecule (Lutheran blood group) [Source:HGNC Symbol;Acc:6722]
BCAR1	10.0	0.003	breast cancer anti-estrogen resistance 1 [Source:HGNC Symbol;Acc:971]
BEGAIN	4.2	0.013	brain-enriched guanylate kinase-associated [Source:HGNC Symbol;Acc:24163]
BGN	7.0	0.003	biglycan [Source:HGNC Symbol;Acc:1044]
BIRC5	4.2	0.002	baculoviral IAP repeat containing 5 [Source:HGNC Symbol;Acc:593]
BLVRB	1.7	0.049	biliverdin reductase B (flavin reductase (NADPH)) [Source:HGNC Symbol;Acc:1063]
BMP4	9.1	0.031	bone morphogenetic protein 4 [Source:HGNC Symbol;Acc:1071]
BMPR1B	18.7	0.005	bone morphogenetic protein receptor, type IB [Source:HGNC Symbol;Acc:1077]
C1QA	2.8	0.005	complement component 1, q subcomponent, A chain [Source:HGNC Symbol;Acc:1241]
C1QB	3.0	0.007	complement component 1, q subcomponent, B chain [Source:HGNC Symbol;Acc:1242]
CALD1	2.9	0.006	caldesmon 1 [Source:HGNC Symbol;Acc:1441]
CALU	2.1	0.041	calumenin [Source:HGNC Symbol;Acc:1458]
CAV1	2.9	0.006	caveolin 1, caveolae protein, 22kDa [Source:HGNC Symbol;Acc:1527]
CBS	10.4	0.000	cystathionine-beta-synthase [Source:HGNC Symbol;Acc:1550]
CCDC80	3.2	0.046	coiled-coil domain containing 80 [Source:HGNC Symbol;Acc:30649]
CCNB1	2.4	0.022	cyclin B1 [Source:HGNC Symbol;Acc:1579]
CCT5	1.8	0.014	chaperonin containing TCP1, subunit 5 (epsilon) [Source:HGNC Symbol;Acc:1618]
CD276	3.6	0.019	CD276 molecule [Source:HGNC Symbol;Acc:19137]
CD300E	1.7	0.048	CD300e molecule [Source:HGNC Symbol;Acc:28874]
CD300LB	1.8	0.048	CD300 molecule-like family member b [Source:HGNC Symbol;Acc:30811]
CD63	1.7	0.027	CD63 molecule [Source:HGNC Symbol;Acc:1692]
CD86	1.7	0.046	CD86 molecule [Source:HGNC Symbol;Acc:1705]
CDA	2.0	0.015	cytidine deaminase [Source:HGNC Symbol;Acc:1712]
CDC20	2.2	0.048	cell division cycle 20 [Source:HGNC Symbol;Acc:1723]
CDC25A	3.9	0.025	cell division cycle 25A [Source:HGNC Symbol;Acc:1725]
CDC42EP1	5.6	0.001	CDC42 effector protein (Rho GTPase binding) 1 [Source:HGNC Symbol;Acc:17014]
CDC6	4.6	0.007	cell division cycle 6 [Source:HGNC Symbol;Acc:1744]

CDCP1	4.9	0.003	CUB domain containing protein 1 [Source:HGNC Symbol;Acc:24357]
CDH13	5.0	0.009	cadherin 13, H-cadherin (heart) [Source:HGNC Symbol;Acc:1753]
CDH2	4.7	0.003	cadherin 2, type 1, N-cadherin (neuronal) [Source:HGNC Symbol;Acc:1759]
CDKN1C	2.0	0.014	cyclin-dependent kinase inhibitor 1C (p57, Kip2) [Source:HGNC Symbol;Acc:1786]
CDKN2A	4.6	0.002	cyclin-dependent kinase inhibitor 2A [Source:HGNC Symbol;Acc:1787]
CDR2L	12.4	0.018	cerebellar degeneration-related protein 2-like [Source:HGNC Symbol;Acc:29999]
CDT1	4.1	0.027	chromatin licensing and DNA replication factor 1 [Source:HGNC Symbol;Acc:24576]
CEBPA	1.9	0.035	CCAAT/enhancer binding protein (C/EBP), alpha [Source:HGNC Symbol;Acc:1833]
CENPF	2.2	0.024	centromere protein F, 350/400kDa [Source:HGNC Symbol;Acc:1857]
CES1	1.8	0.039	carboxylesterase 1 [Source:HGNC Symbol;Acc:1863]
CFP	1.6	0.046	complement factor properdin [Source:HGNC Symbol;Acc:8864]
CGA	4.8	0.041	glycoprotein hormones, alpha polypeptide [Source:HGNC Symbol;Acc:1885]
CLDN1	3.7	0.031	claudin 1 [Source:HGNC Symbol;Acc:2032]
CNRIP1	5.9	0.030	cannabinoid receptor interacting protein 1 [Source:HGNC Symbol;Acc:24546]
COL12A1	3.3	0.003	collagen, type XII, alpha 1 [Source:HGNC Symbol;Acc:2188]
COL1A1	7.5	0.000	collagen, type I, alpha 1 [Source:HGNC Symbol;Acc:2197]
COL3A1	4.5	0.000	collagen, type III, alpha 1 [Source:HGNC Symbol;Acc:2201]
COL4A1	4.9	0.008	collagen, type IV, alpha 1 [Source:HGNC Symbol;Acc:2202]
COL4A2	10.1	0.000	collagen, type IV, alpha 2 [Source:HGNC Symbol;Acc:2203]
COL7A1	16.5	0.000	collagen, type VII, alpha 1 [Source:HGNC Symbol;Acc:2214]
COX6C	1.8	0.027	cytochrome c oxidase subunit VIc [Source:HGNC Symbol;Acc:2285]
COX7B	2.4	0.001	cytochrome c oxidase subunit VIIb [Source:HGNC Symbol;Acc:2291]
COX7C	1.7	0.027	cytochrome c oxidase subunit VIIc [Source:HGNC Symbol;Acc:2292]
CPA4	5.4	0.002	carboxypeptidase A4 [Source:HGNC Symbol;Acc:15740]
CREB3L1	4.6	0.012	cAMP responsive element binding protein 3-like 1 [Source:HGNC Symbol;Acc:18856]
CSDA	1.6	0.041	cold shock domain protein A [Source:HGNC Symbol;Acc:2428]
CSTA	3.2	0.000	cystatin A (stefin A) [Source:HGNC Symbol;Acc:2481]
CTGF	4.7	0.011	connective tissue growth factor [Source:HGNC Symbol;Acc:2500]
CYC1	1.8	0.020	cytochrome c-1 [Source:HGNC Symbol;Acc:2579]

CYR61	6.1	0.001	cysteine-rich, angiogenic inducer, 61 [Source:HGNC Symbol;Acc:2654]
DHRS4	3.1	0.048	dehydrogenase/reductase (SDR family) member 4 [Source:HGNC Symbol;Acc:16985]
DIO2	6.5	0.002	deiodinase, iodothyronine, type II [Source:HGNC Symbol;Acc:2884]
DLX1	7.3	0.022	distal-less homeobox 1 [Source:HGNC Symbol;Acc:2914]
DSG2	2.6	0.040	desmoglein 2 [Source:HGNC Symbol;Acc:3049]
DYNLL1	1.9	0.012	dynein, light chain, LC8-type 1 [Source:HGNC Symbol;Acc:15476]
EDF1	1.9	0.007	endothelial differentiation-related factor 1 [Source:HGNC Symbol;Acc:3164]
EDIL3	3.1	0.005	EGF-like repeats and discoidin I-like domains 3 [Source:HGNC Symbol;Acc:3173]
EFEMP1	4.3	0.006	EGF containing fibulin-like extracellular matrix protein 1 [Source:HGNC Symbol;Acc:3218]
EGFR	4.1	0.005	epidermal growth factor receptor [Source:HGNC Symbol;Acc:3236]
EHD2	5.1	0.003	EH-domain containing 2 [Source:HGNC Symbol;Acc:3243]
ELN	14.2	0.002	elastin [Source:HGNC Symbol;Acc:3327]
EMP1	3.0	0.009	epithelial membrane protein 1 [Source:HGNC Symbol;Acc:3333]
ENAH	2.6	0.044	enabled homolog (Drosophila) [Source:HGNC Symbol;Acc:18271]
EPAS1	2.5	0.031	endothelial PAS domain protein 1 [Source:HGNC Symbol;Acc:3374]
EPHA2	3.7	0.012	EPH receptor A2 [Source:HGNC Symbol;Acc:3386]
EXO1	3.6	0.045	exonuclease 1 [Source:HGNC Symbol;Acc:3511]
EYA4	3.5	0.036	eyes absent homolog 4 (Drosophila) [Source:HGNC Symbol;Acc:3522]
FAM114A1	4.5	0.026	family with sequence similarity 114, member A1 [Source:HGNC Symbol;Acc:25087]
FAM129B	2.1	0.003	family with sequence similarity 129, member B [Source:HGNC Symbol;Acc:25282]
FAM228B	4.3	0.030	family with sequence similarity 228, member B [Source:HGNC Symbol;Acc:24736]
FAM83H	2.8	0.015	family with sequence similarity 83, member H [Source:HGNC Symbol;Acc:24797]
FBLN1	5.7	0.001	fibulin 1 [Source:HGNC Symbol;Acc:3600]
FBN1	3.0	0.011	fibrillin 1 [Source:HGNC Symbol;Acc:3603]
FBN2	2.2	0.014	fibrillin 2 [Source:HGNC Symbol;Acc:3604]
FBP1	2.1	0.011	fructose-1,6-bisphosphatase 1 [Source:HGNC Symbol;Acc:3606]
FCER1A	2.2	0.019	Fc fragment of IgE, high affinity I, receptor for; alpha polypeptide [Source:HGNC Symbol;Acc:3609]
FCN1	1.8	0.031	ficolin (collagen/fibrinogen domain containing) 1 [Source:HGNC Symbol;Acc:3623]
FGF2	3.0	0.050	fibroblast growth factor 2 (basic) [Source:HGNC Symbol;Acc:3676]

FJX1	5.5	0.038	four jointed box 1 (Drosophila) [Source:HGNC Symbol;Acc:17166]
FKBP10	3.6	0.008	FK506 binding protein 10, 65 kDa [Source:HGNC Symbol;Acc:18169]
FLNC	7.5	0.000	filamin C, gamma [Source:HGNC Symbol;Acc:3756]
FN1	4.0	0.000	fibronectin 1 [Source:HGNC Symbol;Acc:3778]
FOLR1	4.7	0.043	folate receptor 1 (adult) [Source:HGNC Symbol;Acc:3791]
FOSB	2.0	0.037	FBJ murine osteosarcoma viral oncogene homolog B [Source:HGNC Symbol;Acc:3797]
FOXM1	2.5	0.007	forkhead box M1 [Source:HGNC Symbol;Acc:3818]
FOXQ1	6.4	0.049	forkhead box Q1 [Source:HGNC Symbol;Acc:20951]
FSCN1	3.0	0.044	fascin homolog 1, actin-bundling protein (Strongylocentrotus purpuratus) [Source:HGNC Symbol;Acc:11148]
FSTL1	2.8	0.018	follistatin-like 1 [Source:HGNC Symbol;Acc:3972]
GABRE	3.2	0.031	gamma-aminobutyric acid (GABA) A receptor, epsilon [Source:HGNC Symbol;Acc:4085]
GADD45GIP1	3.1	0.012	growth arrest and DNA-damage-inducible, gamma interacting protein 1 [Source:HGNC Symbol;Acc:29996]
GAR1	2.6	0.039	GAR1 ribonucleoprotein homolog (yeast) [Source:HGNC Symbol;Acc:14264]
GDF15	6.8	0.014	growth differentiation factor 15 [Source:HGNC Symbol;Acc:30142]
GNA15	1.8	0.034	guanine nucleotide binding protein (G protein), alpha 15 (Gq class) [Source:HGNC Symbol;Acc:4383]
GNG12	2.6	0.030	guanine nucleotide binding protein (G protein), gamma 12 [Source:HGNC Symbol;Acc:19663]
GPC1	5.0	0.004	glypican 1 [Source:HGNC Symbol;Acc:4449]
GPNMB	4.7	0.030	glycoprotein (transmembrane) nmb [Source:HGNC Symbol;Acc:4462]
GPRC5A	3.6	0.005	G protein-coupled receptor, family C, group 5, member A [Source:HGNC Symbol;Acc:9836]
GPRC5C	2.9	0.040	G protein-coupled receptor, family C, group 5, member C [Source:HGNC Symbol;Acc:13309]
GREM1	4.8	0.007	gremlin 1 [Source:HGNC Symbol;Acc:2001]
GSTO1	1.7	0.036	glutathione S-transferase omega 1 [Source:HGNC Symbol;Acc:13312]
GTF2IRD1	5.1	0.014	GTF2I repeat domain containing 1 [Source:HGNC Symbol;Acc:4661]
HBA1	2.3	0.002	hemoglobin, alpha 1 [Source:HGNC Symbol;Acc:4823]
HBD	1.8	0.031	hemoglobin, delta [Source:HGNC Symbol;Acc:4829]
HBG1	5.7	0.002	hemoglobin, gamma A [Source:HGNC Symbol;Acc:4831]
HES1	4.6	0.022	hairy and enhancer of split 1, (Drosophila) [Source:HGNC Symbol;Acc:5192]
HMOX1	1.8	0.020	heme oxygenase (decycling) 1 [Source:HGNC Symbol;Acc:5013]
HSPB8	7.1	0.022	heat shock 22kDa protein 8 [Source:HGNC Symbol;Acc:30171]

HTRA1	2.9	0.037	HtrA serine peptidase 1 [Source:HGNC Symbol;Acc:9476]
ID1	3.0	0.027	inhibitor of DNA binding 1, dominant negative helix-loop-helix protein [Source:HGNC Symbol;Acc:5360]
IER5L	4.4	0.004	immediate early response 5-like [Source:HGNC Symbol;Acc:23679]
IFI6	1.8	0.030	interferon, alpha-inducible protein 6 [Source:HGNC Symbol;Acc:4054]
IGFBP4	2.7	0.002	insulin-like growth factor binding protein 4 [Source:HGNC Symbol;Acc:5473]
IGFBP6	3.6	0.050	insulin-like growth factor binding protein 6 [Source:HGNC Symbol;Acc:5475]
IGFN1	7.3	0.000	immunoglobulin-like and fibronectin type III domain containing 1 [Source:HGNC Symbol;Acc:24607]
IQGAP3	3.6	0.017	IQ motif containing GTPase activating protein 3 [Source:HGNC Symbol;Acc:20669]
ISG15	1.9	0.029	ISG15 ubiquitin-like modifier [Source:HGNC Symbol;Acc:4053]
ITGA11	3.7	0.008	integrin, alpha 11 [Source:HGNC Symbol;Acc:6136]
ITGA3	2.8	0.001	integrin, alpha 3 (antigen CD49C, alpha 3 subunit of VLA-3 receptor) [Source:HGNC Symbol;Acc:6139]
JUND	1.6	0.042	jun D proto-oncogene [Source:HGNC Symbol;Acc:6206]
KCNK1	5.1	0.023	potassium channel, subfamily K, member 1 [Source:HGNC Symbol;Acc:6272]
KIF18B	2.7	0.034	kinesin family member 18B [Source:HGNC Symbol;Acc:27102]
KIF2C	3.4	0.039	kinesin family member 2C [Source:HGNC Symbol;Acc:6393]
KIRREL	4.3	0.008	kin of IRRE like (Drosophila) [Source:HGNC Symbol;Acc:15734]
KLF10	1.7	0.033	Kruppel-like factor 10 [Source:HGNC Symbol;Acc:11810]
KLF11	1.8	0.028	Kruppel-like factor 11 [Source:HGNC Symbol;Acc:11811]
KRT18	6.1	0.012	keratin 18 [Source:HGNC Symbol;Acc:6430]
KRT7	9.7	0.000	keratin 7 [Source:HGNC Symbol;Acc:6445]
KRT8	13.5	0.002	keratin 8 [Source:HGNC Symbol;Acc:6446]
KRT80	5.3	0.003	keratin 80 [Source:HGNC Symbol;Acc:27056]
L1CAM	4.3	0.003	L1 cell adhesion molecule [Source:HGNC Symbol;Acc:6470]
LACE1	5.3	0.050	lactation elevated 1 [Source:HGNC Symbol;Acc:16411]
LAIR2	9.1	0.000	leukocyte-associated immunoglobulin-like receptor 2 [Source:HGNC Symbol;Acc:6478]
LAMA3	8.2	0.035	laminin, alpha 3 [Source:HGNC Symbol;Acc:6483]
LAMA5	2.2	0.014	laminin, alpha 5 [Source:HGNC Symbol;Acc:6485]
LAMB1	4.9	0.024	laminin, beta 1 [Source:HGNC Symbol;Acc:6486]
LAMC1	2.4	0.004	laminin, gamma 1 (formerly LAMB2) [Source:HGNC Symbol;Acc:6492]

LAMTOR2	2.0	0.018	late endosomal/lysosomal adaptor, MAPK and MTOR activator 2 [Source:HGNC Symbol;Acc:29796]
LGALS1	2.2	0.002	lectin, galactoside-binding, soluble, 1 [Source:HGNC Symbol;Acc:6561]
LGALS2	1.8	0.022	lectin, galactoside-binding, soluble, 2 [Source:HGNC Symbol;Acc:6562]
LIF	3.2	0.034	leukemia inhibitory factor [Source:HGNC Symbol;Acc:6596]
LIMCH1	5.0	0.001	LIM and calponin homology domains 1 [Source:HGNC Symbol;Acc:29191]
LMNA	3.2	0.000	lamin A/C [Source:HGNC Symbol;Acc:6636]
LRFN4	2.4	0.040	leucine rich repeat and fibronectin type III domain containing 4 [Source:HGNC Symbol;Acc:28456]
LSM10	1.8	0.032	LSM10, U7 small nuclear RNA associated [Source:HGNC Symbol;Acc:17562]
LURAP1L	3.8	0.038	leucine rich adaptor protein 1-like [Source:HGNC Symbol;Acc:31452]
LY6K	6.8	0.001	lymphocyte antigen 6 complex, locus K [Source:HGNC Symbol;Acc:24225]
MAGEC1	15.5	0.008	melanoma antigen family C, 1 [Source:HGNC Symbol;Acc:6812]
MAP1B	2.5	0.019	microtubule-associated protein 1B [Source:HGNC Symbol;Acc:6836]
MARCKS	1.9	0.016	myristoylated alanine-rich protein kinase C substrate [Source:HGNC Symbol;Acc:6759]
MCAM	3.2	0.010	melanoma cell adhesion molecule [Source:HGNC Symbol;Acc:6934]
MCM4	2.6	0.030	minichromosome maintenance complex component 4 [Source:HGNC Symbol;Acc:6947]
MDK	2.9	0.043	midkine (neurite growth-promoting factor 2) [Source:HGNC Symbol;Acc:6972]
MGST1	2.0	0.025	microsomal glutathione S-transferase 1 [Source:HGNC Symbol;Acc:7061]
MGST2	2.3	0.032	microsomal glutathione S-transferase 2 [Source:HGNC Symbol;Acc:7063]
MIB2	2.1	0.016	mindbomb E3 ubiquitin protein ligase 2 [Source:HGNC Symbol;Acc:30577]
MKI67	2.0	0.009	antigen identified by monoclonal antibody Ki-67 [Source:HGNC Symbol;Acc:7107]
MMP2	3.3	0.016	matrix metallopeptidase 2 (gelatinase A, 72 kDa gelatinase, 72 kDa type IV collagenase) [Source:HGNC Symbol;Acc:7166]
MRPL27	2.6	0.015	mitochondrial ribosomal protein L27 [Source:HGNC Symbol;Acc:14483]
MRPL36	2.4	0.044	mitochondrial ribosomal protein L36 [Source:HGNC Symbol;Acc:14490]
MRPL52	2.1	0.030	mitochondrial ribosomal protein L52 [Source:HGNC Symbol;Acc:16655]
MRPL54	3.0	0.012	mitochondrial ribosomal protein L54 [Source:HGNC Symbol;Acc:16685]
MRPS15	1.8	0.033	mitochondrial ribosomal protein S15 [Source:HGNC Symbol;Acc:14504]
MRPS24	1.8	0.044	mitochondrial ribosomal protein S24 [Source:HGNC Symbol;Acc:14510]
MRPS34	1.8	0.027	mitochondrial ribosomal protein S34 [Source:HGNC Symbol;Acc:16618]

MS4A4A	2.5	0.019	membrane-spanning 4-domains, subfamily A, member 4A [Source:HGNC Symbol;Acc:13371]
MSRB1	1.8	0.029	methionine sulfoxide reductase B1 [Source:HGNC Symbol;Acc:14133]
MT2A	1.8	0.028	metallothionein 2A [Source:HGNC Symbol;Acc:7406]
MYADM	1.6	0.040	myeloid-associated differentiation marker [Source:HGNC Symbol;Acc:7544]
MYBL2	2.1	0.015	v-myb myeloblastosis viral oncogene homolog (avian)-like 2 [Source:HGNC Symbol;Acc:7548]
MYEOV2	2.1	0.025	myeloma overexpressed 2 [Source:HGNC Symbol;Acc:21314]
MYL9	1.9	0.006	myosin, light chain 9, regulatory [Source:HGNC Symbol;Acc:15754]
MYO10	4.3	0.002	myosin X [Source:HGNC Symbol;Acc:7593]
NACA2	3.2	0.026	nascent polypeptide-associated complex alpha subunit 2 [Source:HGNC Symbol;Acc:23290]
NCL	1.9	0.045	nucleolin [Source:HGNC Symbol;Acc:7667]
NDUFA7	2.2	0.025	NADH dehydrogenase (ubiquinone) 1 alpha subcomplex, 7, 14.5kDa [Source:HGNC Symbol;Acc:7691]
NDUFAB1	2.4	0.006	NADH dehydrogenase (ubiquinone) 1, alpha/beta subcomplex, 1, 8kDa [Source:HGNC Symbol;Acc:7694]
NDUFS8	2.1	0.012	NADH dehydrogenase (ubiquinone) Fe-S protein 8, 23kDa (NADH-coenzyme Q reductase) [Source:HGNC Symbol;Acc:7715]
NFATC4	4.0	0.009	nuclear factor of activated T-cells, cytoplasmic, calcineurin-dependent 4 [Source:HGNC Symbol;Acc:7778]
NNMT	6.2	0.004	nicotinamide N-methyltransferase [Source:HGNC Symbol;Acc:7861]
NOL7	2.0	0.034	nucleolar protein 7, 27kDa [Source:HGNC Symbol;Acc:21040]
NOP10	1.7	0.037	NOP10 ribonucleoprotein [Source:HGNC Symbol;Acc:14378]
NOTCH3	3.2	0.032	notch 3 [Source:HGNC Symbol;Acc:7883]
NPB	2.0	0.021	neuropeptide B [Source:HGNC Symbol;Acc:30099]
NPR3	6.8	0.004	natriuretic peptide receptor C/guanylate cyclase C (atrionatriuretic peptide receptor C) [Source:HGNC Symbol;Acc:7945]
NQO1	4.5	0.000	NAD(P)H dehydrogenase, quinone 1 [Source:HGNC Symbol;Acc:2874]
NR2F2	3.8	0.027	nuclear receptor subfamily 2, group F, member 2 [Source:HGNC Symbol;Acc:7976]
NR4A1	1.7	0.047	nuclear receptor subfamily 4, group A, member 1 [Source:HGNC Symbol;Acc:7980]
NRP1	3.7	0.028	neuropilin 1 [Source:HGNC Symbol;Acc:8004]
NUPR1	8.6	0.017	nuclear protein, transcriptional regulator, 1 [Source:HGNC Symbol;Acc:29990]
PACSIN3	8.2	0.001	protein kinase C and casein kinase substrate in neurons 3 [Source:HGNC Symbol;Acc:8572]
PAPPA	3.5	0.020	pregnancy-associated plasma protein A, pappalysin 1 [Source:HGNC Symbol;Acc:8602]

PAPSS2	2.4	0.033	3'-phosphoadenosine 5'-phosphosulfate synthase 2 [Source:HGNC Symbol;Acc:8604]
PARK7	1.7	0.034	parkinson protein 7 [Source:HGNC Symbol;Acc:16369]
PDE2A	3.2	0.009	phosphodiesterase 2A, cGMP-stimulated [Source:HGNC Symbol;Acc:8777]
PDE3A	2.6	0.047	phosphodiesterase 3A, cGMP-inhibited [Source:HGNC Symbol;Acc:8778]
PDLIM7	1.8	0.028	PDZ and LIM domain 7 (enigma) [Source:HGNC Symbol;Acc:22958]
PHGDH	3.4	0.002	phosphoglycerate dehydrogenase [Source:HGNC Symbol;Acc:8923]
PHLDA2	3.6	0.042	pleckstrin homology-like domain, family A, member 2 [Source:HGNC Symbol;Acc:12385]
PHLDA3	4.6	0.022	pleckstrin homology-like domain, family A, member 3 [Source:HGNC Symbol;Acc:8934]
PHLDB1	5.0	0.011	pleckstrin homology-like domain, family B, member 1 [Source:HGNC Symbol;Acc:23697]
PHPT1	1.9	0.016	phosphohistidine phosphatase 1 [Source:HGNC Symbol;Acc:30033]
PID1	1.9	0.049	phosphotyrosine interaction domain containing 1 [Source:HGNC Symbol;Acc:26084]
PIEZO2	7.4	0.027	piezo-type mechanosensitive ion channel component 2 [Source:HGNC Symbol;Acc:26270]
PLB1	2.3	0.030	phospholipase B1 [Source:HGNC Symbol;Acc:30041]
PLS3	3.0	0.050	plastin 3 [Source:HGNC Symbol;Acc:9091]
PMEL	11.6	0.022	premelanosome protein [Source:HGNC Symbol;Acc:10880]
PNP	1.7	0.023	purine nucleoside phosphorylase [Source:HGNC Symbol;Acc:7892]
PODXL	3.4	0.007	podocalyxin-like [Source:HGNC Symbol;Acc:9171]
POLR2F	2.5	0.013	polymerase (RNA) II (DNA directed) polypeptide F [Source:HGNC Symbol;Acc:9193]
POLR2L	2.3	0.002	polymerase (RNA) II (DNA directed) polypeptide L, 7.6kDa [Source:HGNC Symbol;Acc:9199]
POSTN	4.0	0.010	periostin, osteoblast specific factor [Source:HGNC Symbol;Acc:16953]
PPAP2C	5.4	0.019	phosphatidic acid phosphatase type 2C [Source:HGNC Symbol;Acc:9230]
PPARG	18.8	0.010	peroxisome proliferator-activated receptor gamma [Source:HGNC Symbol;Acc:9236]
PPBP	1.9	0.006	pro-platelet basic protein (chemokine (C-X-C motif) ligand 7) [Source:HGNC Symbol;Acc:9240]
PRAME	4.2	0.027	preferentially expressed antigen in melanoma [Source:HGNC Symbol;Acc:9336]
PRDX1	1.8	0.016	peroxiredoxin 1 [Source:HGNC Symbol;Acc:9352]
PSMA6	2.1	0.015	proteasome (prosome, macropain) subunit, alpha type, 6 [Source:HGNC Symbol;Acc:9535]
PSMB5	1.8	0.038	proteasome (prosome, macropain) subunit, beta type, 5 [Source:HGNC Symbol;Acc:9542]
PSMD4	2.0	0.017	proteasome (prosome, macropain) 26S subunit, non-ATPase, 4 [Source:HGNC Symbol;Acc:9561]
PTAFR	1.7	0.030	platelet-activating factor receptor [Source:HGNC Symbol;Acc:9582]

PTGES	4.7	0.004	prostaglandin E synthase [Source:HGNC Symbol;Acc:9599]
PTGFRN	2.8	0.037	prostaglandin F2 receptor inhibitor [Source:HGNC Symbol;Acc:9601]
PTPRF	3.3	0.015	protein tyrosine phosphatase, receptor type, F [Source:HGNC Symbol;Acc:9670]
PTRF	4.4	0.000	polymerase I and transcript release factor [Source:HGNC Symbol;Acc:9688]
PTTG1	2.6	0.048	pituitary tumor-transforming 1 [Source:HGNC Symbol;Acc:9690]
PXDN	4.1	0.002	peroxidasin homolog (Drosophila) [Source:HGNC Symbol;Acc:14966]
RAB34	1.9	0.039	RAB34, member RAS oncogene family [Source:HGNC Symbol;Acc:16519]
RAB3IL1	5.5	0.018	RAB3A interacting protein (rabin3)-like 1 [Source:HGNC Symbol;Acc:9780]
RASIP1	3.3	0.039	Ras interacting protein 1 [Source:HGNC Symbol;Acc:24716]
RND3	5.5	0.002	Rho family GTPase 3 [Source:HGNC Symbol;Acc:671]
ROBO1	5.2	0.040	roundabout, axon guidance receptor, homolog 1 (Drosophila) [Source:HGNC Symbol;Acc:10249]
ROMO1	3.1	0.014	reactive oxygen species modulator 1 [Source:HGNC Symbol;Acc:16185]
RPL14	1.8	0.017	ribosomal protein L14 [Source:HGNC Symbol;Acc:10305]
RPL19	1.9	0.012	ribosomal protein L19 [Source:HGNC Symbol;Acc:10312]
RPL22L1	3.3	0.010	ribosomal protein L22-like 1 [Source:HGNC Symbol;Acc:27610]
RPL23A	1.8	0.021	ribosomal protein L23a [Source:HGNC Symbol;Acc:10317]
RPL24	1.7	0.038	ribosomal protein L24 [Source:HGNC Symbol;Acc:10325]
RPL26	2.3	0.001	ribosomal protein L26 [Source:HGNC Symbol;Acc:10327]
RPL27	2.3	0.001	ribosomal protein L27 [Source:HGNC Symbol;Acc:10328]
RPL27A	1.8	0.015	ribosomal protein L27a [Source:HGNC Symbol;Acc:10329]
RPL29	2.1	0.002	ribosomal protein L29 [Source:HGNC Symbol;Acc:10331]
RPL30	1.8	0.022	ribosomal protein L30 [Source:HGNC Symbol;Acc:10333]
RPL32	2.4	0.008	ribosomal protein L32 [Source:HGNC Symbol;Acc:10336]
RPL34	1.8	0.020	ribosomal protein L34 [Source:HGNC Symbol;Acc:10340]
RPL35	2.6	0.000	ribosomal protein L35 [Source:HGNC Symbol;Acc:10344]
RPL37	2.9	0.012	ribosomal protein L37 [Source:HGNC Symbol;Acc:10347]
RPL37A	2.6	0.006	ribosomal protein L37a [Source:HGNC Symbol;Acc:10348]
RPL38	1.9	0.010	ribosomal protein L38 [Source:HGNC Symbol;Acc:10349]
RPS11	1.9	0.040	ribosomal protein S11 [Source:HGNC Symbol;Acc:10384]

RPS12	1.7	0.038	ribosomal protein S12 [Source:HGNC Symbol;Acc:10385]
RPS15	1.7	0.036	ribosomal protein S15 [Source:HGNC Symbol;Acc:10388]
RPS19	3.2	0.000	ribosomal protein S19 [Source:HGNC Symbol;Acc:10402]
RPS27	1.7	0.030	ribosomal protein S27 [Source:HGNC Symbol;Acc:10416]
RPS7	1.8	0.036	ribosomal protein S7 [Source:HGNC Symbol;Acc:10440]
RPS8	1.9	0.014	ribosomal protein S8 [Source:HGNC Symbol;Acc:10441]
RRP12	1.8	0.021	ribosomal RNA processing 12 homolog (S. cerevisiae) [Source:HGNC Symbol;Acc:29100]
S100A10	1.8	0.011	S100 calcium binding protein A10 [Source:HGNC Symbol;Acc:10487]
S100A12	1.7	0.033	S100 calcium binding protein A12 [Source:HGNC Symbol;Acc:10489]
S100A16	8.9	0.001	S100 calcium binding protein A16 [Source:HGNC Symbol;Acc:20441]
S100A2	8.8	0.007	S100 calcium binding protein A2 [Source:HGNC Symbol;Acc:10492]
S100A8	2.0	0.011	S100 calcium binding protein A8 [Source:HGNC Symbol;Acc:10498]
S100A9	3.2	0.001	S100 calcium binding protein A9 [Source:HGNC Symbol;Acc:10499]
SCARA3	6.5	0.005	scavenger receptor class A, member 3 [Source:HGNC Symbol;Acc:19000]
SEMA3F	6.4	0.039	sema domain, immunoglobulin domain (Ig), short basic domain, secreted, (semaphorin) 3F [Source:HGNC Symbol;Acc:10728]
SERPINE1	3.2	0.012	serpin peptidase inhibitor, clade E (nexin, plasminogen activator inhibitor type 1), member 1 [Source:HGNC Symbol;Acc:8583]
SERPINH1	3.5	0.000	serpin peptidase inhibitor, clade H (heat shock protein 47), member 1, (collagen binding protein 1) [Source:HGNC Symbol;Acc:1546]
SFN	3.7	0.011	stratifin [Source:HGNC Symbol;Acc:10773]
SH3BP4	2.6	0.022	SH3-domain binding protein 4 [Source:HGNC Symbol;Acc:10826]
SH3D19	3.7	0.021	SH3 domain containing 19 [Source:HGNC Symbol;Acc:30418]
SIX2	8.7	0.035	SIX homeobox 2 [Source:HGNC Symbol;Acc:10888]
SLC3A2	1.8	0.030	solute carrier family 3 (activators of dibasic and neutral amino acid transport), member 2 [Source:HGNC Symbol;Acc:11026]
SLC6A9	3.5	0.041	solute carrier family 6 (neurotransmitter transporter, glycine), member 9 [Source:HGNC Symbol;Acc:11056]
SLC7A11	2.1	0.012	solute carrier family 7 (anionic amino acid transporter light chain, xc- system), member 11 [Source:HGNC Symbol;Acc:11059]

SLC7A5	2.1	0.003	solute carrier family 7 (amino acid transporter light chain, L system), member 5 [Source:HGNC Symbol;Acc:11063]
SLC9A3R2	3.4	0.019	solute carrier family 9, subfamily A (NHE3, cation proton antiporter 3), member 3 regulator 2 [Source:HGNC Symbol;Acc:11076]
SMOC1	3.0	0.030	SPARC related modular calcium binding 1 [Source:HGNC Symbol;Acc:20318]
SOD1	2.0	0.004	superoxide dismutase 1, soluble [Source:HGNC Symbol;Acc:11179]
SPAG7	1.8	0.049	sperm associated antigen 7 [Source:HGNC Symbol;Acc:11216]
SPI1	1.8	0.022	spleen focus forming virus (SFFV) proviral integration oncogene spi1 [Source:HGNC Symbol;Acc:11241]
SPINK6	6.5	0.048	serine peptidase inhibitor, Kazal type 6 [Source:HGNC Symbol;Acc:29486]
SPOCK1	3.3	0.006	sparc/osteonectin, cwcv and kazal-like domains proteoglycan (testican) 1 [Source:HGNC Symbol;Acc:11251]
SPRED1	4.0	0.025	sprouty-related, EVH1 domain containing 1 [Source:HGNC Symbol;Acc:20249]
ST14	1.8	0.046	suppression of tumorigenicity 14 (colon carcinoma) [Source:HGNC Symbol;Acc:11344]
STAB1	1.9	0.047	stabilin 1 [Source:HGNC Symbol;Acc:18628]
STC2	5.5	0.000	stanniocalcin 2 [Source:HGNC Symbol;Acc:11374]
STRA6	11.0	0.005	stimulated by retinoic acid 6 [Source:HGNC Symbol;Acc:30650]
TEAD1	2.6	0.046	TEA domain family member 1 (SV40 transcriptional enhancer factor) [Source:HGNC Symbol;Acc:11714]
TEAD4	6.0	0.025	TEA domain family member 4 [Source:HGNC Symbol;Acc:11717]
TGFB1I1	3.0	0.022	transforming growth factor beta 1 induced transcript 1 [Source:HGNC Symbol;Acc:11767]
TIMP3	3.2	0.033	TIMP metallopeptidase inhibitor 3 [Source:HGNC Symbol;Acc:11822]
TMSB10	1.9	0.027	thymosin beta 10 [Source:HGNC Symbol;Acc:11879]
TNFRSF12A	4.3	0.025	tumor necrosis factor receptor superfamily, member 12A [Source:HGNC Symbol;Acc:18152]
TNNI2	3.6	0.020	troponin I type 2 (skeletal, fast) [Source:HGNC Symbol;Acc:11946]
TPD52L1	4.0	0.027	tumor protein D52-like 1 [Source:HGNC Symbol;Acc:12006]
TPM1	2.4	0.002	tropomyosin 1 (alpha) [Source:HGNC Symbol;Acc:12010]
TPM2	2.6	0.001	tropomyosin 2 (beta) [Source:HGNC Symbol;Acc:12011]
TREM1	2.3	0.005	triggering receptor expressed on myeloid cells 1 [Source:HGNC Symbol;Acc:17760]
TRIM16L	4.2	0.026	tripartite motif containing 16-like [Source:HGNC Symbol;Acc:32670]
TRIP6	2.3	0.025	thyroid hormone receptor interactor 6 [Source:HGNC Symbol;Acc:12311]
TRNP1	5.2	0.035	TMF1-regulated nuclear protein 1 [Source:HGNC Symbol;Acc:34348]
TSKU	5.3	0.005	tsukushi, small leucine rich proteoglycan [Source:HGNC Symbol;Acc:28850]

TUBA1A	2.1	0.004	tubulin, alpha 1a [Source:HGNC Symbol;Acc:20766]
TUBA1B	1.9	0.007	tubulin, alpha 1b [Source:HGNC Symbol;Acc:18809]
TUBB2A	4.9	0.020	tubulin, beta 2A class IIa [Source:HGNC Symbol;Acc:12412]
TUBB6	2.7	0.022	tubulin, beta 6 class V [Source:HGNC Symbol;Acc:20776]
UBL5	3.6	0.000	ubiquitin-like 5 [Source:HGNC Symbol;Acc:13736]
UCN2	9.4	0.021	urocortin 2 [Source:HGNC Symbol;Acc:18414]
UQCR10	1.8	0.027	ubiquinol-cytochrome c reductase, complex III subunit X [Source:HGNC Symbol;Acc:30863]
UQCRFS1	2.1	0.019	ubiquinol-cytochrome c reductase, Rieske iron-sulfur polypeptide 1 [Source:HGNC Symbol;Acc:12587]
UQCRH	1.7	0.047	ubiquinol-cytochrome c reductase hinge protein [Source:HGNC Symbol;Acc:12590]
UQCRQ	2.1	0.009	ubiquinol-cytochrome c reductase, complex III subunit VII, 9.5kDa [Source:HGNC Symbol;Acc:29594]
USMG5	3.1	0.008	up-regulated during skeletal muscle growth 5 homolog (mouse) [Source:HGNC Symbol;Acc:30889]
VIM	2.0	0.038	vimentin [Source:HGNC Symbol;Acc:12692]
WDR62	2.4	0.027	WD repeat domain 62 [Source:HGNC Symbol;Acc:24502]
WNT5A	3.4	0.006	wingless-type MMTV integration site family, member 5A [Source:HGNC Symbol;Acc:12784]
WNT7B	4.7	0.032	wingless-type MMTV integration site family, member 7B [Source:HGNC Symbol;Acc:12787]
ZNF503	4.5	0.008	zinc finger protein 503 [Source:HGNC Symbol;Acc:23589]
**Genes whose expression decreased after three cycles of oxaliplatin therapy (in alphabetical order)**			
ADAMTS10	−1.7	0.042	ADAM metallopeptidase with thrombospondin type 1 motif, 10 [Source:HGNC Symbol;Acc:13201]
AGAP1	−3.6	0.009	ArfGAP with GTPase domain, ankyrin repeat and PH domain 1 [Source:HGNC Symbol;Acc:16922]
AHSP	−2.1	0.012	alpha hemoglobin stabilizing protein [Source:HGNC Symbol;Acc:18075]
ALOX15B	−7.9	0.002	arachidonate 15-lipoxygenase, type B [Source:HGNC Symbol;Acc:434]
AOC3	−4.1	0.039	amine oxidase, copper containing 3 (vascular adhesion protein 1) [Source:HGNC Symbol;Acc:550]
ATP1A3	−2.5	0.012	ATPase, Na+/K+ transporting, alpha 3 polypeptide [Source:HGNC Symbol;Acc:801]
BRPF3	−1.7	0.047	bromodomain and PHD finger containing, 3 [Source:HGNC Symbol;Acc:14256]
BTLA	−2.3	0.010	B and T lymphocyte associated [Source:HGNC Symbol;Acc:21087]
CACNA1I	−1.7	0.029	calcium channel, voltage-dependent, T type, alpha 1I subunit [Source:HGNC Symbol;Acc:1396]
CAMP	−2.8	0.033	cathelicidin antimicrobial peptide [Source:HGNC Symbol;Acc:1472]
CCL20	−6.7	0.010	chemokine (C-C motif) ligand 20 [Source:HGNC Symbol;Acc:10619]

CCL4	−1.9	0.016	chemokine (C-C motif) ligand 4 [Source:HGNC Symbol;Acc:10630]
CD69	−1.7	0.025	CD69 molecule [Source:HGNC Symbol;Acc:1694]
CDK5R1	−2.4	0.005	cyclin-dependent kinase 5, regulatory subunit 1 (p35) [Source:HGNC Symbol;Acc:1775]
CH25H	−53.3	0.020	cholesterol 25-hydroxylase [Source:HGNC Symbol;Acc:1907]
CHI3L1	−5.3	0.012	chitinase 3-like 1 (cartilage glycoprotein-39) [Source:HGNC Symbol;Acc:1932]
CLEC4E	−1.9	0.014	C-type lectin domain family 4, member E [Source:HGNC Symbol;Acc:14555]
CMPK1	−1.7	0.034	cytidine monophosphate (UMP-CMP) kinase 1, cytosolic [Source:HGNC Symbol;Acc:18170]
CSF3	−51.2	0.023	colony stimulating factor 3 (granulocyte) [Source:HGNC Symbol;Acc:2438]
CXCL1	−8.8	0.038	chemokine (C-X-C motif) ligand 1 (melanoma growth stimulating activity, alpha) [Source:HGNC Symbol;Acc:4602]
CXCL2	−3.4	0.007	chemokine (C-X-C motif) ligand 2 [Source:HGNC Symbol;Acc:4603]
CXCL3	−5.0	0.004	chemokine (C-X-C motif) ligand 3 [Source:HGNC Symbol;Acc:4604]
CXCL5	−2.1	0.049	chemokine (C-X-C motif) ligand 5 [Source:HGNC Symbol;Acc:10642]
DDIT4	−1.8	0.012	DNA-damage-inducible transcript 4 [Source:HGNC Symbol;Acc:24944]
DLEU7	−6.6	0.046	deleted in lymphocytic leukemia, 7 [Source:HGNC Symbol;Acc:17567]
DNAJB14	−1.7	0.043	DnaJ (Hsp40) homolog, subfamily B, member 14 [Source:HGNC Symbol;Acc:25881]
DRAXIN	−3.7	0.018	dorsal inhibitory axon guidance protein [Source:HGNC Symbol;Acc:25054]
DUSP1	−2.0	0.010	dual specificity phosphatase 1 [Source:HGNC Symbol;Acc:3064]
ECHDC3	−5.0	0.010	enoyl CoA hydratase domain containing 3 [Source:HGNC Symbol;Acc:23489]
EGR1	−4.7	0.000	early growth response 1 [Source:HGNC Symbol;Acc:3238]
EGR3	−2.6	0.027	early growth response 3 [Source:HGNC Symbol;Acc:3240]
EMB	−1.7	0.019	embigin [Source:HGNC Symbol;Acc:30465]
EREG	−5.2	0.002	epiregulin [Source:HGNC Symbol;Acc:3443]
ERN1	−2.3	0.001	endoplasmic reticulum to nucleus signaling 1 [Source:HGNC Symbol;Acc:3449]
ETS2	−1.7	0.028	v-ets erythroblastosis virus E26 oncogene homolog 2 (avian) [Source:HGNC Symbol;Acc:3489]
FBXO32	−1.8	0.017	F-box protein 32 [Source:HGNC Symbol;Acc:16731]
FCRL1	−1.7	0.024	Fc receptor-like 1 [Source:HGNC Symbol;Acc:18509]
FCRL6	−1.9	0.006	Fc receptor-like 6 [Source:HGNC Symbol;Acc:31910]
FKBP5	−2.7	0.000	FK506 binding protein 5 [Source:HGNC Symbol;Acc:3721]

FOS	−1.8	0.033	FBJ murine osteosarcoma viral oncogene homolog [Source:HGNC Symbol;Acc:3796]
GAPT	−1.8	0.026	GRB2-binding adaptor protein, transmembrane [Source:HGNC Symbol;Acc:26588]
GFRA1	−17.2	0.000	GDNF family receptor alpha 1 [Source:HGNC Symbol;Acc:4243]
GJB2	−48.9	0.020	gap junction protein, beta 2, 26kDa [Source:HGNC Symbol;Acc:4284]
GPR56	−1.9	0.021	G protein-coupled receptor 56 [Source:HGNC Symbol;Acc:4512]
GPR97	−2.5	0.044	G protein-coupled receptor 97 [Source:HGNC Symbol;Acc:13728]
HCST	−1.9	0.008	hematopoietic cell signal transducer [Source:HGNC Symbol;Acc:16977]
HIST1H4C	−6.6	0.044	histone cluster 1, H4c [Source:HGNC Symbol;Acc:4787]
HLA-DQA2	−2.1	0.034	major histocompatibility complex, class II, DQ alpha 2 [Source:HGNC Symbol;Acc:4943]
HNRNPD	−6.5	0.044	heterogeneous nuclear ribonucleoprotein D (AU-rich element RNA binding protein 1, 37kDa) [Source:HGNC Symbol;Acc:5036]
IGJ	−2.0	0.004	immunoglobulin J polypeptide, linker protein for immunoglobulin alpha and mu polypeptides [Source:HGNC Symbol;Acc:5713]
IKZF3	−1.7	0.026	IKAROS family zinc finger 3 (Aiolos) [Source:HGNC Symbol;Acc:13178]
IL1A	−12.1	0.012	interleukin 1, alpha [Source:HGNC Symbol;Acc:5991]
IL1B	−4.2	0.000	interleukin 1, beta [Source:HGNC Symbol;Acc:5992]
IL6	−7.0	0.000	interleukin 6 (interferon, beta 2) [Source:HGNC Symbol;Acc:6018]
IL6ST	−1.7	0.042	interleukin 6 signal transducer (gp130, oncostatin M receptor) [Source:HGNC Symbol;Acc:6021]
IL7R	−1.9	0.023	interleukin 7 receptor [Source:HGNC Symbol;Acc:6024]
IL8	−14.5	0.000	interleukin 8 [Source:HGNC Symbol;Acc:6025]
INHBA	−19.6	0.001	inhibin, beta A [Source:HGNC Symbol;Acc:6066]
IPCEF1	−1.8	0.019	interaction protein for cytohesin exchange factors 1 [Source:HGNC Symbol;Acc:21204]
KIAA1551	−2.0	0.008	KIAA1551 [Source:HGNC Symbol;Acc:25559]
KIR3DX1	−2.8	0.041	killer cell immunoglobulin-like receptor, three domains, X1 [Source:HGNC Symbol;Acc:25043]
KLF9	−2.0	0.007	Kruppel-like factor 9 [Source:HGNC Symbol;Acc:1123]
KLRD1	−2.0	0.007	killer cell lectin-like receptor subfamily D, member 1 [Source:HGNC Symbol;Acc:6378]
KRT23	−4.1	0.039	keratin 23 (histone deacetylase inducible) [Source:HGNC Symbol;Acc:6438]
LCNL1	−3.3	0.029	lipocalin-like 1 [Source:HGNC Symbol;Acc:34436]
LGR6	−1.8	0.042	leucine-rich repeat containing G protein-coupled receptor 6 [Source:HGNC Symbol;Acc:19719]

LRRN3	−2.0	0.040	leucine rich repeat neuronal 3 [Source:HGNC Symbol;Acc:17200]
LTF	−3.5	0.001	lactotransferrin [Source:HGNC Symbol;Acc:6720]
MAGT1	−1.9	0.017	magnesium transporter 1 [Source:HGNC Symbol;Acc:28880]
MAN1A1	−1.8	0.018	mannosidase, alpha, class 1A, member 1 [Source:HGNC Symbol;Acc:6821]
METTL7A	−1.7	0.021	methyltransferase like 7A [Source:HGNC Symbol;Acc:24550]
MMP25	−1.8	0.049	matrix metallopeptidase 25 [Source:HGNC Symbol;Acc:14246]
MMP9	−6.5	0.001	matrix metallopeptidase 9 (gelatinase B, 92kDa gelatinase, 92kDa type IV collagenase) [Source:HGNC Symbol;Acc:7176]
MS4A3	−3.7	0.025	membrane-spanning 4-domains, subfamily A, member 3 (hematopoietic cell-specific) [Source:HGNC Symbol;Acc:7317]
MYLIP	−1.8	0.023	myosin regulatory light chain interacting protein [Source:HGNC Symbol;Acc:21155]
NEU4	−5.0	0.027	sialidase 4 [Source:HGNC Symbol;Acc:21328]
NPEPPS	−1.6	0.050	aminopeptidase puromycin sensitive [Source:HGNC Symbol;Acc:7900]
NR3C2	−2.0	0.032	nuclear receptor subfamily 3, group C, member 2 [Source:HGNC Symbol;Acc:7979]
NUDT16	−1.7	0.024	nudix (nucleoside diphosphate linked moiety X)-type motif 16 [Source:HGNC Symbol;Acc:26442]
PDK4	−2.2	0.006	pyruvate dehydrogenase kinase, isozyme 4 [Source:HGNC Symbol;Acc:8812]
PIGV	−2.1	0.022	phosphatidylinositol glycan anchor biosynthesis, class V [Source:HGNC Symbol;Acc:26031]
PIK3IP1	−1.7	0.036	phosphoinositide-3-kinase interacting protein 1 [Source:HGNC Symbol;Acc:24942]
PKP2	−6.4	0.005	plakophilin 2 [Source:HGNC Symbol;Acc:9024]
PLEKHG3	−2.1	0.024	pleckstrin homology domain containing, family G (with RhoGef domain) member 3 [Source:HGNC Symbol;Acc:20364]
POU2AF1	−1.8	0.021	POU class 2 associating factor 1 [Source:HGNC Symbol;Acc:9211]
PRDM1	−2.1	0.017	PR domain containing 1, with ZNF domain [Source:HGNC Symbol;Acc:9346]
PRKAB2	−1.7	0.036	protein kinase, AMP-activated, beta 2 non-catalytic subunit [Source:HGNC Symbol;Acc:9379]
PRRX1	−6.5	0.003	paired related homeobox 1 [Source:HGNC Symbol;Acc:9142]
PTGDR	−2.2	0.032	prostaglandin D2 receptor (DP) [Source:HGNC Symbol;Acc:9591]
PTGS2	−5.2	0.000	prostaglandin-endoperoxide synthase 2 (prostaglandin G/H synthase and cyclooxygenase) [Source:HGNC Symbol;Acc:9605]
RAB7L1	−1.7	0.038	RAB7, member RAS oncogene family-like 1 [Source:HGNC Symbol;Acc:9789]

RASD2	−21.8	0.011	RASD family, member 2 [Source:HGNC Symbol;Acc:18229]
RNF165	−2.9	0.048	ring finger protein 165 [Source:HGNC Symbol;Acc:31696]
RNF4	−1.6	0.048	ring finger protein 4 [Source:HGNC Symbol;Acc:10067]
SERPINB2	−38.5	0.000	serpin peptidase inhibitor, clade B (ovalbumin), member 2 [Source:HGNC Symbol;Acc:8584]
SESN1	−2.7	0.000	sestrin 1 [Source:HGNC Symbol;Acc:21595]
SGCA	−12.0	0.040	sarcoglycan, alpha (50kDa dystrophin-associated glycoprotein) [Source:HGNC Symbol;Acc:10805]
SLAMF6	−1.7	0.022	SLAM family member 6 [Source:HGNC Symbol;Acc:21392]
SLC1A3	−13.3	0.000	solute carrier family 1 (glial high affinity glutamate transporter), member 3 [Source:HGNC Symbol;Acc:10941]
SLC22A1	−6.8	0.012	solute carrier family 22 (organic cation transporter), member 1 [Source:HGNC Symbol;Acc:10963]
SLC4A7	−1.8	0.016	solute carrier family 4, sodium bicarbonate cotransporter, member 7 [Source:HGNC Symbol;Acc:11033]
SMAP2	−1.8	0.042	small ArfGAP2 [Source:HGNC Symbol;Acc:25082]
SOCS3	−1.6	0.039	suppressor of cytokine signaling 3 [Source:HGNC Symbol;Acc:19391]
SOD2	−15.7	0.020	superoxide dismutase 2, mitochondrial [Source:HGNC Symbol;Acc:11180]
SPRY1	−7.6	0.002	sprouty homolog 1, antagonist of FGF signaling (Drosophila) [Source:HGNC Symbol;Acc:11269]
TBX15	−6.0	0.018	T-box 15 [Source:HGNC Symbol;Acc:11594]
TCL1A	−2.1	0.004	T-cell leukemia/lymphoma 1A [Source:HGNC Symbol;Acc:11648]
THBS1	−3.9	0.000	thrombospondin 1 [Source:HGNC Symbol;Acc:11785]
TTC9	−1.8	0.047	tetratricopeptide repeat domain 9 [Source:HGNC Symbol;Acc:20267]
TTLL10	−18.8	0.029	tubulin tyrosine ligase-like family, member 10 [Source:HGNC Symbol;Acc:26693]
UBA52	−2.0	0.039	ubiquitin A-52 residue ribosomal protein fusion product 1 [Source:HGNC Symbol;Acc:12458]
USP28	−1.7	0.039	ubiquitin specific peptidase 28 [Source:HGNC Symbol;Acc:12625]
USP9Y	−2.0	0.036	ubiquitin specific peptidase 9, Y-linked [Source:HGNC Symbol;Acc:12633]
XBP1	−1.7	0.025	X-box binding protein 1 [Source:HGNC Symbol;Acc:12801]
YPEL1	−4.0	0.024	yippee-like 1 (Drosophila) [Source:HGNC Symbol;Acc:12845]
ZBTB38	−1.7	0.033	zinc finger and BTB domain containing 38 [Source:HGNC Symbol;Acc:26636]
